# A Narrative Review on Current Status of Conscious Sedation for Transcatheter Aortic Valve Implantation

**DOI:** 10.3390/medicina61111980

**Published:** 2025-11-05

**Authors:** Georgia Nazou, Nikolaos Schizas, Konstantina N. Romana, Vasiliki Androutsopoulou, Eleni Magira, Andreas Sarantopoulos, Dimitrios Iliopoulos, Spyros D. Mentzelopoulos

**Affiliations:** 1 Anaesthesiology Department, Evangelismos General Hospital, 10676 Athens, Greece; georgianazou@gmail.com (G.N.); conromana@yahoo.gr (K.N.R.); 2 School of Medicine, National and Kapodistrian University of Athens, 12462 Athens, Greecesdmentzelopoulos@yahoo.com (S.D.M.); 3 4th Cardiac Surgery Department, Hygeia Hospital, 15123 Athens, Greece; 4 Department of Cardiothoracic Surgery, University Hospital of Larissa, 41110 Larissa, Greece; androutsopoulouvasiliki@hotmail.com; 5 1st Department of Critical Care and Pulmonary Services, Evangelismos General Hospital, 10676 Athens, Greece; 6 NCH Rooney Heart Institute, Naples, FL 34102, USA; andreas.sarantopoulos01@gmail.com

**Keywords:** Transcatheter Aortic Valve Implantation/Replacement, conscious sedation, Anesthesiologic management, aortic valve stenosis, general anesthesia

## Abstract

Anesthesiologic management of Transcatheter Aortic Valve Implantation (TAVI) is a key factor in procedural success and effectiveness. Although general anesthesia was the main anesthesiologic approach during the early years of the development of TAVI, over the last decade, there has been a shift towards sedation. Hemodynamic stability is the main concern of intraoperative anesthesiologic management. Preprocedural, multidisciplinary assessment of the patient is essential prior to TAVI and should include a full anesthesiologic evaluation. TAVI offers a number of advantages to patients and medical teams, but important accompanying complications and anesthesiologic risks remain. In this narrative review, all aspects of sedation in TAVI are presented and analyzed, including methods, patient selection, contraindications, drug administration, intraprocedural parameters, outcomes, and future developments in this field.

## 1. Introduction

The journey of Transcatheter Aortic Valve Implantation (TAVI) is entering its third decade since its first application [1]. Indeed, indicators for TAVI are being expanded to younger and lower-risk patients, although this technique was initially developed solely for high-risk patients over the age of 75. According to the new ESC/EACTS guidelines for valvular disease (2025), the age threshold for TAVI consideration is now 70 years old [2]. This shift to lower age limits is anticipated to further increase the number of patients with aortic valve stenosis treated with TAVI as the annual growth rate is estimated to be 4–10%, with more than 300,000 implants per year in Europe and North America [3,4].

In parallel with the evolution of transcatheter techniques, anesthesiologic management in this field is also developing. Initially, general anesthesia (GA) was the principal choice, but after the first decade of TAVI implementation, several centers began using conscious sedation (CS) as an alternative. Evidence from comparative studies favors the use of CS over GA, as this approach has come to be associated with improved clinical outcomes [5]. Nowadays, the anesthesiologic management of TAVI patients depends on the practice of each center, as data from questionnaires have revealed great heterogeneity among various centers throughout Europe [6]. In any case, many scientists believe that sedation should be the main anesthesiologic strategy in all cases, with a few exceptions, such as those where access for TAVI is not transfemoral [7]. Indeed, they highlight the fact that CS can be applied to 90–95% of TAVI patients [7].

In this narrative review, all aspects of CS will be presented, including preoperative evaluation, patient preference, anesthetic considerations, intraoperative management, postoperative observation, and clinical outcomes.

## 2. Methods

This is a narrative review based on a search of PubMed, Embase, Cohrane and Google Scholar from database inception through September and October 2025 by two reviewers. The search combined the terms “Transcatheter Aortic Valve Implantation/Replacement” or “TAVI” or “Aortic valve stenosis” with “anesthesiologic management,” or “sedation” or “general anesthesia,” without date restrictions. Peer-reviewed, English-language articles were considered if they described peri- or intra-operative uses and outcomes of CS in TAVI procedures. Eligible evidence included observational studies, randomized comparative trials, prospective studies, reviews, and meta-analyses directly addressing anesthesiologic management with CS in TAVI patients.

Titles and abstracts were screened by two reviewers to identify relevant reports; full texts were then assessed for inclusion. From each article, we extracted the study design, model or patient cohort, type of anesthesia, medications administered, and key procedural techniques or clinical outcomes. Because this was not a systematic review and the included studies were heterogeneous in design, endpoints, and platforms, we did not perform risk-of-bias assessments or meta-analysis. Findings were organized into thematic domains and synthesized qualitatively (Table 1).

## 3. Results

**Figure 1:**
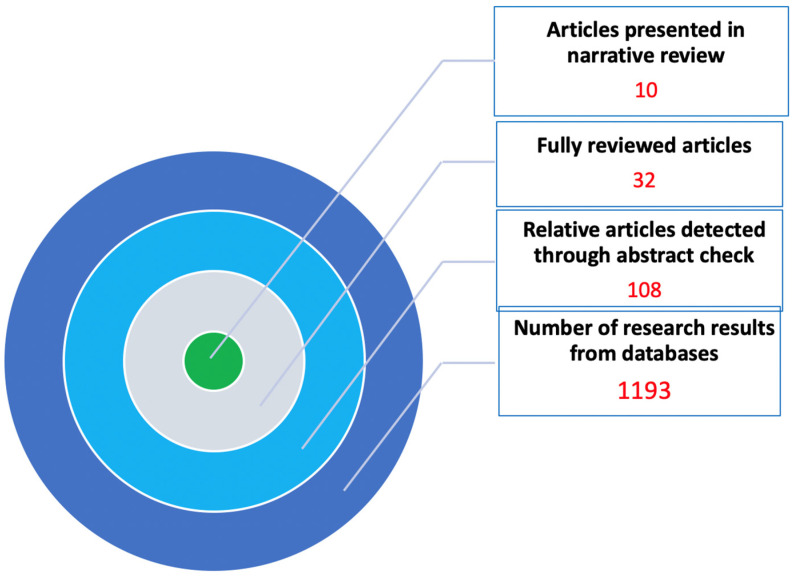
Flow diagram representing the article’s selection process.

During the literature review on CS for TAVI, 108 publications with high relevance were found. These articles constitute the pool of data from which the evidence for this narrative review was derived. As mentioned before, a systematic review and/or meta-analysis was not feasible due to great heterogeneity among the published studies on methods, design, drugs and intraprocedural techniques studied and presentation of outcomes. Therefore, articles of recent well-designed studies including a satisfactory number of patients are presented analytically to facilitate the study on the current status of sedation in patients submitted to TAVI. In total, 10 articles from the last 5 years were chosen and are presented in Table 1. The process of article selection is presented in Table 2 and Figure 1. (Table 2, Figure 1) These articles were chosen due to their novelty, scientific soundness, and clinical significance, in order to assess the current trends and hot topics in this field.

The subjects and the design of the studies regarding CS in TAVI vary a great deal. Most of the studies are retrospective and comparative with matched populations. (Table 1) At least three randomized comparative trials were detected in this field, with two of them being multicenter. The studies aimed to compare anesthesiologic approaches in TAVI patients and the general categories were (1) comparison of CS with GA (three studies); (2) comparison of various CS methods (four studies); (3) comparison of CS with local anesthesia (LA) (two studies); and (4) investigation of the impact of virtual reality (VR) (1 study).

As regards the comparison between GA and CS, the outcomes favored CS. More specifically, CS was associated with improved hemodynamic stability and lower complication rates [11], reduced ICU and hospital stay [11,12], lower costs [12], and possibly lower all-cause mortality [13]. It should be mentioned that the number of patients included in these studies is satisfactory in terms of supporting the aforementioned conclusions.

Four studies compared various CS methods. Kinoshita et al. investigated the impact of iliaca fascia ropivacaine administration added to CS and found that the TAVI patients treated with this technique experienced less pain [9]. A comparative study on CS with Dexmedetomidine (DEX) and remimazolam showed that the latter resulted in better recovery findings [10]. On the other hand, another study compared CS with remimazolam versus the combination of DEX and propofol and observed no statistically significant difference [16]. CS with ketamine combined with DEX versus ketamine combined with propofol was investigated in a really interesting systematic review and meta-analysis [14]. This research favored ketamine combined with DEX in both adult and pediatric populations at high risk of nausea and vomiting [14]. In this domain, many CS strategies are being implemented, and more are also being developed over time as new drugs are administered in sedation.

LA is an anesthesiologic approach that has emerged over the past years. Two studies comparing LA to CS are included in Table 1. The clinical outcomes of LA and CS are similar, although a recent study showed that the experience of anxiety was higher for patients in the LA group [8,17]. One study investigated the impact of virtual reality on TAVI patients, but this showed neither benefits nor drawbacks [15].

## 4. Discussion

Anesthesiologic management is constantly evolving, and many types of scientifically sound studies are being conducted and published. With the exception of studies comparing GA to CS, the studies investigating sedation approaches present a high degree of heterogeneity in terms of their study designs and the exact methods used. This study is a narrative review and, in the following paragraphs, several parameters regarding SC will be presented and discussed.

### 4.1. Is Anesthesiologist-Led Anesthesia Necessary for TAVI?

A number of articles investigating anesthesia led by cardiologists and nurses have been published in recent years. Cardiologist-led anesthesia showed efficacy and safety during TAVI in two recent studies in selected patients [18,19]. In the same vein, a study evaluating the efficiency of nurse-led monitoring during sedation in TAVI patients found safe and satisfactory outcomes [20]. On the other hand, monitored anesthesia with anesthesiologists presented superior clinical outcomes in a large meta-analysis including data from more than 45,000 patients [21]. Anesthesiologic management in TAVI patients should not be neglected, as these procedures still present serious complications. Data from a European registry showed that an anesthesiologist always participated in TAVI procedures in 73% of centers and in more than half of such procedures in 12% of the centers [22]. We strongly recommend anesthesiologist-led anesthesia in any case in such procedures, although some evidence supports the fact that nurse-led or cardiologist-led anesthesia is safe in selected patients.

### 4.2. Type of Anesthesia Selection

The choice between GA and SC depends on the patient’s characteristics, the common practice of each center, and the decision of each anesthesiologist. A recent report from the European TAVI Pathway found that SC is used in 60% of cases, LA was chosen in 33% of cases, and that only 7% of the patients were treated with GA [22]. This survey may underestimate the percentage of patients receiving GA and overestimate the number of patients receiving only LA in “real-world” practice, but it reflects the trend towards SC combined with LA.

More than 40 studies investigated various parameters regarding anesthesia type during TAVI, and 3 of them are included in Table 1 [11,12,13]. A meta-analysis found that CS can reduce the length of hospitalization, procedural time, 30-day mortality, and cardiovascular drug administration, while there was no statistical difference detected between general anesthesia and sedation in permanent pacemaker placement, cardiogenic shock, myocardial infarction, acute kidney injury, and procedural effectiveness [23]. SOLVE-TAVI was a randomized trial, including 447 patients undergoing TAVI. The results of the study suggest that the primary composite endpoint (consisting of mortality, stroke, myocardial infarction, infection requiring antibiotic treatment, and renal failure) occurred in 51.4% in the CS group and 61.3% for the GA group in 5 years follow up, while all-cause mortality was lower for CS group compared to GA (41.5% vs. 54.3%; HR: 0.70; 95% CI: 0.53–0.94; *p* = 0.02) [13]. Another review of 13 studies with data on 6718 patients indicated that the outcomes remain similar between the two groups after one year [24]. In the same vein, studies have also shown that there is no difference regarding acute kidney injury and neurocognitive outcomes [25,26].

On the other hand, a randomized study performed between 2014 and 2018 in two centers, consisting of data on 477 consecutive patients, suggests that the CS group was associated with higher efficiency compared to the GA group, while the safety was similar for both groups [26]. More specifically, reduced length of stay (2 versus 3 days, *p* < 0.001), inotropes (13% vs. 32%, *p* < 0.001), blood transfusions (10% vs. 22%, *p* < 0.0008), contrast volume (50 vs. 90 mL, *p* < 0.001), and fluoroscopy time (20 vs. 24 min, *p* < 0.001) were found for the CS group compared to GA group, respectively. According to the authors of this study, these findings support the superiority of CS over GA [26]. A comparative study between CS and GA found that three-quarters of those patients presenting side effects were from the GA group [27]. A study on a sample of 107 patients found that GA was related to a significantly higher rate of admission to an intensive care unit, acute kidney injury, and the new onset of atrial fibrillation [28]. Additionally, data show that GA is associated with a higher rate of pulmonary complications (21.4% for GA versus 3.3% for CS) in TAVI patients with chronic obstructive pulmonary disease [29]. Moreover, a systematic review of a large cohort showed that the risk of postoperative delirium was significantly higher in patients aged over 75 years who were treated with GA when compared to CS [30]. Another study, in which 204 patients were studied, showed that the CS group received less catecholamines and less intravenous fluid during the procedure, while the conversion rate ranged from 3.1% to 4.6% [31,32].

There is much evidence from numerous publications in this field that indicates that both strategies are acceptable. Indeed, a sub-study of the SOLVE-TAVI trial administered a self-evaluated well-being and satisfaction questionnaire and came to the conclusion that no statistically significant difference was detected [33]. The choice of one method or the other is, in practice, mainly determined by the anesthesiologist and patient preference, but more randomized trials may more clearly define the criteria that should be considered in order to meet each patient’s needs [34].

**Figure 2:**
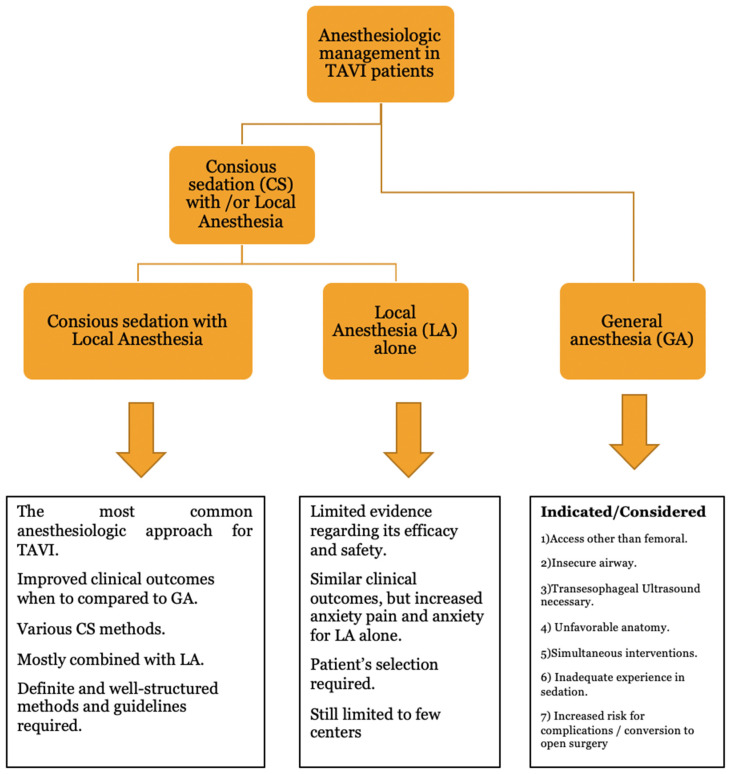
Algorithm presenting the anesthesiologic approaches in TAVI patients.

Although the choice of the type of anesthesia still mainly relies on the preference of each physician, there are several criteria that significantly impact this choice (see Figure 2). Femoral access, favorable anatomic characteristics (e.g., good femoral artery or aorta anatomy, aortic root anatomy, and calcification of the aortic valve), stable patient hemodynamics, no anticipated problems with the airway, short procedural time expected, and no need for transesophageal echo (TEE) are the factors that favor the choice of CS combined with LA [35]. There are also several conditions in which GA is the safest approach, including the following: (1) patient other than femoral (etc. transapical, transcarotid, transaortic, or transubclavian); (2) insecure airway; (3) requirement for TEE; (4) increased risk for intraprocedural complications (unfavorable anatomic, TAVI-in-TAVI, and small aortic annulus); (5) prolonged procedural time expected; (6) extreme agitation or frailty; (7) inadequate experience or an early stage of the learning curve for TAVI operators; and (8) simultaneous concomitant procedures (etc. transcatheter endovascular aortic repair) [36,37]. The criteria are still relatively general and the choices in clinical practice are mostly made based on the physician or the center protocol and the level of experience.

### 4.3. Conscious Sedation (SC) with Local Anesthesia (LA)

CS in combination with local anesthesia in the access site is presently the most common anesthesiologic approach in clinical practice. Monitoring during TAVI procedures conducted using CS is similar to that using GA. Standard monitoring includes an electrocardiogram, pulse oximetry, capnography, invasive blood pressure monitoring, and a Venturi mask that delivers a controlled percentage of oxygen.

As regards LA, lidocaine hydrochloride 2% solution is injected into the access site; femoral access is usually preferred by cardiologists or cardiac surgeons. Alternatively, other local anesthetics such as ropivacaine may be used [9]. In addition to this, iliaca fascia or femoral nerve blocks are used in some centers in order to minimize sedation requirements [35].

CS is achieved by the intravenous administration of drugs, including but not limited to propofol infusion, midazolam, remifentanil, ketamine, remimazolam, and dexmedetomidine. The dosage and rate of administration of the drugs, as well as their combined administration, should be individualized and titrated to attain the desired result. Indicative dosages and interactions for each drug are presented in Table 3. The anesthesiologist should take into consideration the age of the patient, the degree of aortic valve stenosis, the patient’s ASA score, comorbidities, and any previous interventional procedures, in order to perform CS and modify the dosages accordingly. The objective is to secure hemodynamic stability and to be aware of complications such as bleeding and arrhythmia. In addition, it is important to have access to ventilation throughout the procedure and to be aware of possible conversion to GA, as its incidence ranges between 3% and 8% [38,39].

The current trend in the anesthesiologic management of TAVI patients is to perform CS with LA when anatomic characteristics are favorable and the patient is hemodynamically stable [40]. Although there is no definite algorithm for CS in TAVI patients, most authors suggest that sedation medication is at the discretion of the anesthesiologist and the hospital protocol [41]. In this sense, the SC method varies considerably among centers, and the preferred medication is highly related to the level of familiarity of each physician with a sedation drug.

DEX alone or more often with another drug, such as remifentanil, is widely used. Despite the fact that DEX administration is associated with bradycardia, the majority of anesthesiologists choose this drug for CS in short procedures [42]. DEX is a highly selective alpha-2 agonist with sedative and analgesic properties that maintains spontaneous breathing. Patients can be awakened by stimulation, while remaining calm and contactable [43]. DEX alone during TAVI preserves gas exchange, maintains hemodynamic stability, decreases postoperative delirium, and provides a deep level of sedation safely [43]. Indeed, DEX is associated with lower values of PaCO_2_ and reduced requirements for vasopressor support when compared to a combination of propofol and opioids in TAVI patients [44]. However, the level of analgesia is moderate and, therefore, it is usually administered with other medications such as opioids, propofol, or ketamine. As mentioned before, CS with ketamine combined with DEX versus ketamine combined with propofol has been found to carry a higher risk of nausea and vomiting in both adult and pediatric populations [14].

There is a growing number of published articles studying the efficacy of remimazolam in TAVI patients. A study of 464 patients found that remimazolam presented a reduced need for temporary pacing and vassopressors when compared to DEX, suggesting better recovery hemodynamics for remimazolam [10]. No statistically significant differences between these two medications were observed in a recent smaller cohort of 177 patients [16]. More data are anticipated regarding remimazolam in order to delineate its efficacy in TAVI patients.

### 4.4. Special Considerations

Procedural risks for TAVI have dramatically improved, but still 1,3% of the cases require open-heart surgery due to valve migration, aortic annulus rupture, dissection, cardiac tamponade, and other factors [45]. These complications require urgent surgical intervention, and the implementation of cardiopulmonary bypass (CPB) may be necessary. Moreover, the CPB machine and cardiac surgeon must be on standby for the treatment of such complications. During the procedure and after its completion, there is a high incidence of vascular complications, which reaches 17.3% for major complications, and all are correlated to increased 30-day mortality [46].

Another serious complication after TAVI is stroke, with a rate of 3.3% despite the fact that the subclinical incidence of cerebral infarction is very high. It is of great significance to highlight that the patient should be closely monitored during the procedure for signs of stroke and that CS offers a clear advantage in this field [47]. Additionally, heart rate disturbances are very common in these patients, and when high-risk factors for rate disturbances are present, an intraprocedural management plan should be initiated [48].

As has been mentioned, CS presents several benefits for TAVI patients and is also feasible for most patients. On the other hand, the cases where CS may increase the risk of intraprocedural events should be identified and, when required, the GA approach should be used from the beginning. In technically difficult cases in which TEE or patient control may be necessary, GA is the preferred method [49]. Some parameters that make TEE necessary are a small aortic annulus, extensive atheromatosis in the aortic annulus, TAVI in TAVI, and when significant paravalvular leakage is present or anticipated. Another point of great importance is airway management. As the conversion rate ranges around 5%, emergent intubation is relatively common. Difficult intubation in a hemodynamically unstable patient during TAVI can be fatal; therefore, such cases should receive extra care and attention. Factors such as extreme obesity, limited neck extension, limited mouth opening, and previous regional surgery or irradiation should be evaluated accordingly [50]. Moreover, the presence of bleeding due to heparin administration or other secretions makes the intubation more difficult. It should be noted that ventilation methods such as high-flow nasal oxygen do not improve oxygenation in such patients [51]. During the procedure, heart disturbances may occur, and when present, a plan for their management should be made. As an example, a large study including 198 patients with left bundle branch block after TAVI found that this condition did not impact 1-year survival [52]. Therefore, even if the standard practice is SC with LA, factors such as the difficulty of the procedure or the management of the airway should not be neglected at the preoperative planning stage.

### 4.5. Future Developments

A series of randomized control trials, such as SURTAVI, NOTION-2, PARTNER-3, Evolut Low Risk and others, are now in progress and will provide useful data on the safety and efficacy of TAVI in moderate- and low-risk patients [53]. Hence, TAVI may expand to younger and lower-risk patients, which may also impact anesthesiologic management. At the same time, the use of CS in clinical practice is constantly being evaluated by retrospective and prospective studies; therefore, more evidence helping to standardize methods and indications will become available in the future. Moreover, there is a gap in knowledge regarding patient—reported outcomes, such as anxiety and satisfaction, and future studies should investigate these parameters. Additionally, new drugs are also being developed, which will undoubtedly impact CS for TAVI. We also anticipate with great interest new applications of LA and VR in clinical practice. To sum up, bearing in mind the evolution of CS for TAVI over the last decade, significant developments are expected in the years to come.

### 4.6. Limitations

This is a narrative rather than a systematic review as the existing studies are mostly heterogenous in design, methods, medications studied, and primary end-points. Additionally, the scope of this study is not to compare two CS approaches but to investigate the current status of CS for TAVI and present useful information on its management. The articles presented in Table 1 were chosen by the authors not to align with certain criteria, but as articles with scientific soundness that represent the main hot research topics in this domain.

## 5. Conclusions

The scientific interest in the domain of anesthesiologic management in TAVI patients, as evidenced by the number of published articles and the application of new methods or medications, is increasing. There are no definite criteria regarding the selection of anesthesia type, and the methods used in SC vary considerably. Anesthesiologist-led anesthesia is fundamental and results in better periprocedural clinical outcomes. Currently, SC with LA is the main anesthesiologic approach, but these choices should be patient-centered rather than strictly adhering to the protocols used in each center.

## Figures and Tables

**Table 1:** Articles published within the last 5 years in which the current trends and hot topics are presented.

Ref	First Author, Year	Study Type	Anesthetic Approach	Number of Patients	Key Findings	Conclusions
[8]	Berkovitch, 2022	Comparative, retrospective, propensity score analysis	Comparison LA to SC	1096	Higher AKI and left bundle branch block for SC Higher in-hospital and 1-year mortality for SC	LA—only strategy is associated with lower complication rates
[9]	Kinoshita, 2022	RCT	SC (DEX combined with RM or PRO) alone or with ropivacaine FIB	72	Reduced frequency of pain and less RM administered for FIB group Reduced cold sensation on the anterior surface of thigh for FIB group	Improved quality of CS for FIB group due to less pain experienced
[10]	Kim, 2024	Comparative, retrospective, propensity score analysis, non-inferiority	Comparison of SC with DEX and remimazolam	464	Time to recovery was similar for the two groups Vassopressor/inotrope administration favored remimazolam group Higher rate of temporary pacemaker requirement for DEX group	Remimazolam was associated with better recovery findings
[11]	Georgia, 2024	Comparative, retrospective	Comparison of GA to SC	102	Increased odds of elevated mean arterial pressure for GA Less anesthesia time, total procedural time and norandrenaline administration for SC More complications (including AKI and respiratory failure) and prolonged ICU and hospital stay for GA	SC was associated with better hemodynamic stability during the procedure, fewer complications, and reduced ICU and hospital stay
[12]	Luzzi, 2024	Comparative, retrospective, matched population	Comparison GA to SC	248	No difference in postoperative mortality and morbidity detected Hospital stay 5 for SC versus 7 for GA Higher total cost of stay for GA group	SC is related to shorter hospital stays and lower costs when compared to GA
[13]	Feistritzer, 2024	Multicenter RCT	Comparison of GA to SC	447	Composite primary endpoint (all-cause mortality, stroke, myocardial infraction, and AKI) was better for SC versus GA (51.4% vs. 61.3%, *p* = 0.09) Lower all-cause mortality for SC (41.5% vs. 54.3%, *p* = 0.02)	Similar clinical outcomes for both groups, although SC may be associated with lower all-cause mortality
[14]	Elsaeidy, 2024	Systematic review and meta-analysis	Comparison between Ketadex* and Ketofol**	1429	Ketadex was associated with lower pain scores, hypoxic events, agitations, and airway obstructions Ketofol presented shorter recovery time, lower incidence of nausea and vomiting, while physician satisfaction was better for this group	Ketadex is better combination for periprocedural SC for both adult and pediatric patients who are not at high risk for nausea and vomit
[15]	Chatterjee, 2025	Single center RCT	Comparison between LA alone versus LA + VR	75	Procedural satisfaction, pain perception, and change in anxiety were not affected by VR use	VR implementation did not affect the experienced pain and anxiety
[16]	Mino, 2025	Comparative, retrospective, propensity score analysis	Comparison SC with Remimazolam versus DEX + PR	177	The incidence of postoperative nausea and vomiting was 4.92% for remimazolam and 3.28% for DEX+PR (*p* = 0.817)	No significant difference between the two groups regarding remimazolam and DEX+PR
[17]	Feistritzer, 2025	Multicenter, RCT, non-inferiority trial	Comparison between LA and SC	752	Primary endpoint incidence (including all-cause mortality, vascular and bleeding complications, and infections and neurologic events in 30 days) was 22.9% for LA and 25.8% for SC Reported anxiety and intraprocedural stress were higher for LA group	LA was non-inferior to SC in clinical outcomes and safety. LA is related to higher anxiety during the procedure.

LA: local anesthesia, SC: conscious sedation, AKI: acute kidney injury, RCT: randomized control trial, DEX: Dexmedetomidine, RM: Remifentanil, PR: Propofol, FIB: Fascia Iliaca Block, GA: general anesthesia, ICU: intensive care unit, Ketadex*: sedation with Ketamine and Dexmedetomidine, Ketofol**: sedation with Ketamine and Propofol, VR: virtual reality.

**Table 2:** The methodology used for article selection.

Databases—Sources of Review	Number of Results	Relative Articles	Fully Reviewed Articles	Articles Presented in Narrative Review
**PUBMED**	145	105	22	10
**EMBASE**	76	52	16	3
**COHRANE**	82	67	19	2
**GOOGLE SCHOLAR**	890	108	22	10
**TOTAL**	1193	108	32	10

**Table 3:** Drugs, interactions, and indicative dosages for conscious sedation during TAVI.

DRUGS	BLOOD PRESSURE	HEART RATE	RESPIRATORY DEPRESSION	ANALGESIA	NAUSEA/ VOMITING	SEDATION DOSAGE
PROPOFOL	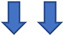	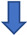	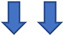	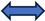	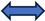	1–2 mg/kg IV for induction 25–150 μg/kg/min infusion
KETAMINE	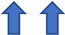	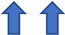	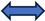	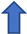	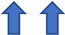	0.5–2 mg/kg IV
DEXDOR	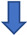	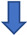	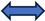	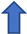	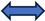	1 μg/kg bolus over 10 min +0.2–0.7 μg/kg/h infusion
MIDAZOLAM	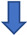	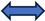	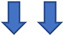	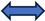	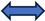	0.02–0.2 mg/kg IV bolus 0.02–0.1 mg/kg/h infusion
REMIMAZOLAM	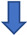	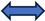	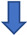	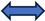	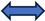	0.1–0.2 mg/kg for induction 1–2 mg/kg/h infusion
FENTANYL	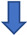	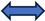	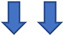	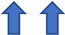	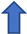	1–2 μg/kg bolus
REMIFENTANIL	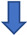	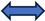	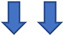	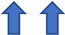	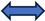	0.5–1 μg/kg bolus 0.05–0.2 μg/kg/min infusion
ETOMIDATE	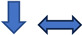	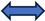	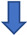	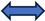	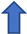	0.2–0.3 mg/kg IV

## Data Availability

The datasets used and/or analyzed during the current study are available from the corresponding author upon reasonable request.
